# Direct Quantitative Analysis of Arsenic in Coal Fly Ash

**DOI:** 10.1155/2012/438701

**Published:** 2012-11-29

**Authors:** Sri Hartuti, Shinji Kambara, Akihiro Takeyama, Kazuhiro Kumabe, Hiroshi Moritomi

**Affiliations:** Department of Environmental and Renewable Energy System, Gifu University, 1-1 Yanagido, Gifu 501-1193, Japan

## Abstract

A rapid, simple method based on graphite furnace atomic absorption spectrometry is described for the direct determination of arsenic in coal fly ash. Solid samples were directly introduced into the atomizer without preliminary treatment. The direct analysis method was not always free of spectral matrix interference, but the stabilization of arsenic by adding palladium nitrate (chemical modifier) and the optimization of the parameters in the furnace program (temperature, rate of temperature increase, hold time, and argon gas flow) gave good results for the total arsenic determination. The optimal furnace program was determined by analyzing different concentrations of a reference material (NIST1633b), which showed the best linearity for calibration. The optimized parameters for the furnace programs for the ashing and atomization steps were as follows: temperatures of 500–1200 and 2150°C, heating rates of 100 and 500°C s^−1^, hold times of 90 and 7 s, and medium then maximum and medium argon gas flows, respectively. The calibration plots were linear with a correlation coefficient of 0.9699. This method was validated using arsenic-containing raw coal samples in accordance with the requirements of the mass balance calculation; the distribution rate of As in the fly ashes ranged from 101 to 119%.

## 1. Introduction

Coal combustion byproducts predominantly consist of fly ash, bottom ash, and boiler slag [1]. The environmental hazards associated with coal combustion byproducts are of concern with respect to potential health effects [2–4]. Among the trace elements in coal fly ashes, arsenic, cadmium, copper, mercury, and lead are the greatest concern as environmental hazards [5].

Arsenic, one of the most highly toxic chemicals, is a semimetallic element commonly found as arsenide and in arsenate compounds. It is an odorless, tasteless, and notoriously poisonous metalloid with many allotropic forms that is dangerous for the environment [6]. Long-term exposure of arsenic contaminated materials to water may lead to various diseases such as conjunctivitis, hyperkeratosis, hyperpigmentation, cardiovascular diseases, disturbance in the peripheral vascular and nervous systems, skin cancer, gangrene, leucomelonisis, nonpitting swelling, hepatomegaly, and splenomegaly [7].

In Japanese coal-fired power plant sites, the ash storage area usually holds seawater and rainwater (excess water); therefore, some elements in the fly ash, including arsenic, are leached out into the excess water. If the arsenic concentration in the excess water exceeds the environmental limit (0.1 mg L^−1^ in Japan), the excess water cannot be drained into the sea. This situation is serious, because ash storage must be discontinued.

Given these concerns, it is important to be able to rapidly determine the arsenic content in the fly ash at these sites. Graphite furnace atomic absorption spectrometry (GFAAS) is one of the most reliable and powerful analytical techniques for the determination of trace elements in water, soil, clinical, and biological samples [8, 9]. It offers good sensitivity with a short analysis time, low cost in comparison with inductively coupled plasma mass spectrometry (ICP-MS) [8], and requires a low sample volume (2–100 *μ*L) [9].

However, most of the reported methods for arsenic determination based on GFAAS require preconcentration, separation [10–13], and dilution steps [10, 13–15]. These steps are disadvantageous with respect to cost and are also time consuming [16]. Therefore, it is necessary to establish a simpler procedure for the accurate determination of arsenic in solids using GFAAS. However, there have been only limited studies on the determination of arsenic using a direct sampling system with solid samples [15, 17, 18].

Therefore, the present paper that focuses on the development of a technique based on GFAAS for the direct measurement of arsenic in several coal fly ashes produced from pulverized-coal-fired boilers. Emphasis was placed on the optimization of the temperature of the furnace program and the use of chemical modifiers to minimize the potential interference.

## 2. Experimental

### 2.1. Instrumentation

A single-beam atomic absorption spectrometer (model novAA400, Analytik Jena) equipped with a monochromator was used for the measurements. This instrument has a Czerny-Turner mount with a plain holographic grating system (1800 lines/mm) that covers the wavelength range from 185 to 900 nm. The spectrometer combines a new transverse-heated graphite furnace atomizer with high-aperture optics and fast background compensation based on an optimized deuterium hollow cathode lamp. The optics system provides efficient background compensation by means of the transillumination of equal absorption volumes for both measurement and correction. Solid and liquid sample introduction modes are possible with a quick change and realignment of the system. For solid samples, an automatic sample weighing system (Sartorius microbalance) and pyrolytically coated graphite tubes with platform boats were used as the sample carriers. 

### 2.2. Standards and Reagents

#### 2.2.1. Construction of the Calibration Curve

Standard samples for the calibration of solid samples (coal fly ash) were prepared from the certified reference material NIST 1633b (Arsenic conc. = 136.2 ± 2.6 mg kg^−1^). The NIST 1633b material was diluted with *α*-Alumina (*α*-Al_2_O_3_) powder to prepare samples of different concentrations. A matrix modifier was prepared by dissolving palladium nitrate (Pd(NO_3_)_2_, 100 ppm) in 40% HNO_3_ and distilled water.

Standard samples for the calibration of liquid samples were prepared from reference solutions of As_2_O_3_ (Merck, pro-analysis). The standard arsenic solutions were prepared in 60% HNO_3_ and distilled water. The matrix modifier was composed of Pd(NO_3_)_2_ (100 ppm) in 3.75% HNO_3_ and distilled water.

#### 2.2.2. Coal Fly Ash Samples

Twenty-one objective samples of coal fly ashes were collected from the electrostatic precipitator in a pulverized-coal-fired power generation process (1000 MWe) using raw coal imported from distinct Indonesian coal mines (Kalimantan Island, Indonesia). To analyze the arsenic concentration in these coal fly ashes, the samples, with added Pd(NO_3_)_2_ (100 ppm), were directly introduced into the GFAAS without any pretreatment (known as direct solid sampling).

To complete the validation of the method, the calculation of the mass balance for arsenic was required. This calculation involved the determination of the arsenic concentration in the raw coal samples.

#### 2.2.3. Pretreatment of Raw Coal Samples

The raw coal samples are the source fuel for the fly ash samples described above. The arsenic concentration in the raw coal samples must be analyzed to validate the accuracy of the determination of the arsenic concentration in the fly ashes. However, because the arsenic concentration in the raw coal samples was too high for GFAAS detection, a dilution process was necessary.

The raw coal samples were prepared through wet destruction procedures using several concentrated acids, followed by heating (200°C), cooling, and filtering processes. The concentrated acids included 60% HNO_3_, 60% H_2_SO_4_, 3.5% HCl, and 30% HF. Other reagents used for the treatment included 2.5% KMnO_4_ and 5%FeCl_3_. Following the wet destruction, the final raw coal samples were obtained in the liquid phase. Five raw coal samples served as the source of the twenty-one coal fly ash samples analyzed in the study. 

### 2.3. Measurement Conditions

The optical parameters used for the direct analysis of arsenic in coal fly ash (solid sampling system) and in the raw coal samples (liquid sampling system) were as follows: wavelength, 193.7 nm; slit width, 1.2 nm; lamp intensity, 6.0 mA. Quantification was carried out by the analysis of the peak area, and pyrolytically coated graphite tubes with platform boats were used for sample introduction.

The optimization sequence for the furnace program used to analyze the different concentrations of the selected certified reference material NIST 1633b is presented in Table 1, and the details of the optimum furnace program developed for the analysis of all of the coal fly ash samples is presented in Table 2. 

With respect to the liquid samples, the optimization sequence for the furnace program used to analyze the reference solutions containing different concentrations of As_2_O_3_ are listed in Table 3, and the details of the optimum furnace program developed for the analysis of all of the raw coal ash samples is presented in Table 4.

The weight of the fly ash samples ranged from approximately 0.5 to 2 mg and was determined using a microbalance. The appropriate amount of the raw coal sample (as a liquid) was determined on the basis of the analyte sensitivity and ranged from approximately 10 to 20 *μ*L. After weighing a sample, the appropriate matrix modifier solution containing palladium nitrate was injected into the sample boat, and the boat was introduced into the furnace. The furnace was then heated according to the specified furnace program and settings for the atomic absorption measurement device.

## 3. Results and Discussion

### 3.1. Optimization of Instrumental Parameters for Solid and Liquid Sample Introduction

There are two wavelengths available for arsenic elemental analysis: 193.7 nm and 197.2 nm. The second most sensitive wavelength for arsenic was selected because of the relatively high arsenic content in the coal fly ash and the certified reference material as determined during the analysis performed using the atomic absorption spectrometry device.

At 193.7 nm, the sample was completely atomized (100% based on the instrument sensitivity), but with high interference, while at 197.2 nm, the sample was only partly atomized (53%) with low interference. To achieve optimum results, analysis at 193.7 nm was selected to ensure that a relatively large quantity of arsenic was available for detection. High interference is associated with the characteristic of arsenic, which has the large difference in the volatility of its compounds, where the oxides are highly volatile, and other compounds are very stable. These properties may lead to analyte loss during pyrolysis and even in the first stage of atomization which made some compounds possible to evaporate together with other elements; therefore, the background signals from other elements in the coal and fly ash increased [19]. To overcome this problem, a chemical modifier (palladium nitrate) was added to reach satisfactory stabilization of arsenic at high temperatures and to reduce the background signals [20]. When the same experiment was repeated, the background wavelengths were not selected, leading to a better signal-to-noise ratio. While this approach is not specific to a particular coal sample, it can be applied in general to all samples. 

The lamp slit makes it possible to adjust the light intensity, which is produced using a hollow cathode lamp. With a high slit, the intensity is high, and with a the low slit, the intensity is low.

The integration time is the length of time during which the atomized sample is in contact with the light passing through it. The integration time for the liquid and solid samples was different because of the difference in properties of the two types of samples.

### 3.2. Development of a Direct Quantitative Analysis Method

To ensure the quality of the analytical results, all of the parameters of each step in the furnace program were optimized, and appropriate calibration graphs were obtained.

The optimization efforts were focused on the pyrolysis/ashing and atomizing steps. The optimum pyrolysis temperature is the maximum temperature at which no losses of the analyte occur, and maximum analyte absorbance and minimum background noise are achieved during atomization. The optimum atomization temperature is the minimum temperature at which a complete and fast evaporation of the analyte is achieved and a reproducible signal (in terms of height and shape of the peak) is recorded. 

For the coal fly ash samples, the experiments was carried out using the optimized furnace program shown in Table 2. The optimization of the furnace program was focused on the ashing and atomization steps. Two previous drying steps were carried out to achieve the correct dryness so that the matrix modifier solvent (distilled water) did not cause splattering. For dry ashing, a high temperature between 500 and 1200°C was used to vaporize any remaining water and other volatile materials and convert any organic substances in the presence of the oxygen in the air to CO_2_, H_2_O, and N_2_ [21].

For each step, the hold time and flow of inert argon gas were studied. In the ashing step, a maximum flow of the inert gas was chosen because it was necessary for the decomposition of the sample matrix to gaseous products. For the atomization step, a temperature of 2150°C was used on the basis of the need to evaporate arsenic, maintain a low-background signal that did not disturb the measurement, and achieve a good level of sensitivity for the signal peak of the analyte. For the liquid sampling system (raw coal sample), the experiments was carried out using the optimized furnace program shown in Table 4. The optimization of this furnace program was also focused on the pyrolysis and atomization steps. Drying steps were again carried out to achieve the correct dryness so that the solvent of the matrix modifier (distilled water) did not cause splattering. For the dry pyrolysis procedure, a high temperature 1500°C was used to vaporize the remaining water and other volatile materials and to convert any organic substances in the presence of the oxygen in air to CO_2_, H_2_O, and N_2_. For each step, the hold time and inert (argon) gas flow rate were studied. In the pyrolysis step, the maximum flow of inert gas was again chosen because it was necessary for the decomposition of the sample matrix to gaseous products. For the atomization step, a temperature of 2500°C was used in this case to achieve a low background signal that did not disturb the measurement and a good level of sensitivity for the signal peak of the analyte.

### 3.3. Establishment of the Quality Parameters for Solid and Liquid Sample Introduction

A calibration curve was constructed by plotting the average peak area against the concentration, and the regression equation was computed. The total absorbance and background signals were recorded during the atomization step of the furnace program when arsenic was evaporated and split from molecules into atoms. The instrument recorded the absorption generated by each given concentration. 

**Figure 1:**
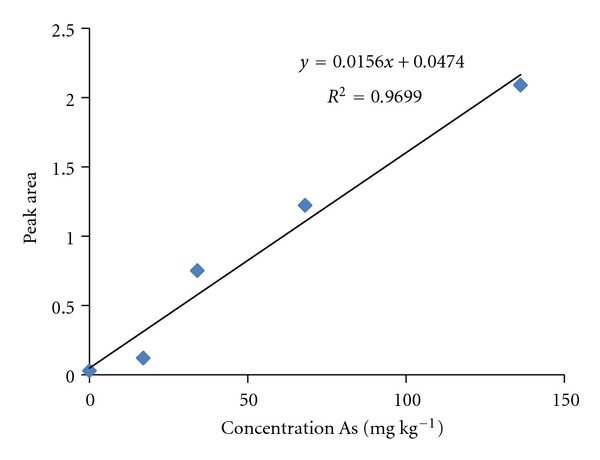
Calibration for solid sample determination by GFAAS.

**Figure 2:**
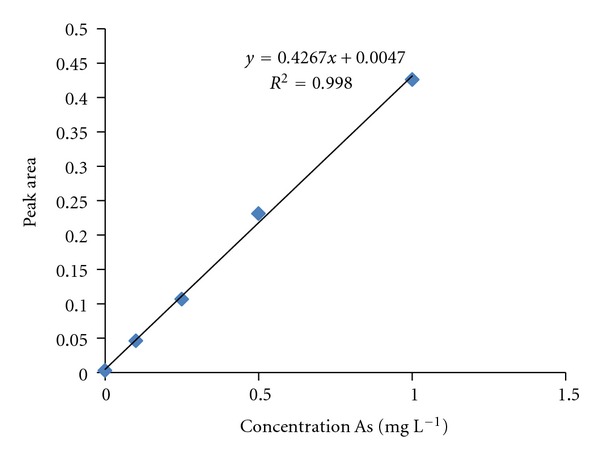
Calibration for liquid sample determination by GFAAS.

For the solid samples, the linearity was measured by analyzing four different concentrations (136.2, 68.1, 34.05, 17.02, and 0 mg kg^−1^) of the certified reference material NIST 1633b. The correlation coefficient (*r*) obtained from the analysis was 0.9699 (Figure 1). For the liquid samples, the linearity was measured by analyzing four different concentrations (10, 2, 1, 0.5, and 0 mg L^−1^) of reference solutions of As_2_O_3_. The correlation coefficient (*r*) obtained from this analysis was 0.9980 (Figure 2).

### 3.4. Quantity of Arsenic in the Coal Fly Ash Samples

The optimized furnace program allowed the determination of arsenic in the coal fly ash and raw coal samples. The obtained results for the analysis of the 21 fly ash and 5 raw coal samples can be seen in Table 5.

The arsenic concentration in the EP 3 samples tended to be greater than that in the EP 2 samples, and the arsenic concentration in the EP 2 samples tended to be greater than that in the EP 1 samples. This trend can be explained by the differences in the particle sizes of the samples; the particle size increased in the order EP 3 < EP 2 < EP 1. The surface area of the smaller particle sizes enables a higher absorption of arsenic in the samples.

The data for the raw coal samples were used to validate the accuracy of the method. The mass balance (distribution rate of arsenic in the coal fly ash) was calculated by the following formula:

(1) $$\begin{array}{c} P_{\text{As}}=\frac{{\text{As}}_{\text{ash}}}{{\text{As}}_{0}^{\prime }}\times 100,\\ {\text{As}}_{0}^{\prime }=\frac{\text{A}{\text{s}}_{0}}{\text{Ash}}\times 100, \end{array}$$

where *P*_As_ is the distribution fraction of arsenic [%], As_ash_ is the average arsenic concentration in the fly ash (EP 1, 2, and 3) [mg kg^−1^-coal, dry basis], As_0_ is the arsenic concentration in the raw coal [mg kg^−1^-coal, dry basis], As_0_′ is the arsenic concentration in the raw coal on an ash basis [mg kg^−1^-coal, dry basis], and Ash is the ash content in the raw coal [%, dry basis].

The results for the mass balance values for each raw coal sample (E, F, G, H, I, J, and K) are shown in Table 6.

## 4. Conclusion 

Based upon the obtained results, we conclude that the direct quantitative analysis of arsenic in solid samples (coal fly ash) is possible using the developed graphite furnace atomic absorption spectroscopy (GF-AAS) method. The determined validation parameters for the developed method are in the commonly acceptable range for this type of analysis, and the good percentages for the mass balance indicate the accuracy of the method. Hence, the proposed method is a simple, accurate, rapid technique that can be employed to routinely determine the amount of arsenic in coal fly ash.

## Figures and Tables

**Table 1:** Optimization of the furnace program for the analysis of coal fly ash (solid sampling system).

Integration time (s)	Ashing temperature(s)	Rate (°C s^−1^)	Hold time (s)	Ar gas (L min^−1^)	Atomizer temperature (°C)	Rate (°C s^−1^)	Hold time (s)	Ar gas (L min^−1^)	Correlation coefficients
10	600	100	20	Max	2100	1000	5	Mid	0.2548
	1200	100	20	Max					

10	600	100	20	Max	2100	1000	5	Mid	0.3879
	1200	100	20	Max					

	500	100	20	Min					
10	1200	100	20	Mid	2000	500	7	Mid	0.5097
	450	100	50	Max					

	500	100	20	Min					
7.5	1200	100	20	Mid	2000	500	7	Mid	0.6584
	450	100	50	Max					

	500	100	20	Min					
6	1200	100	20	Mid	2100	500	7	Mid	0.6641
	450	100	50	Max					

	600	100	20	Max					
5.5	1200	100	20	Mid	2150	500	7	Mid	0.7611
	500	100	50	Max					

	600	100	20	Max					
6	1200	100	20	Mid	2150	500	7	Mid	0.7739
	500	100	50	Max					

	600	100	20	Max					
6	1200	100	20	Mid	2150	500	7	Mid	0.9349
	500	100	50	Max					

Min: 0.1 L min^−1^, Mid: 1.0 L min^−1^, Max: 2.0 L min^−1^.

**Table 2:** Optimized furnace program for the analysis of coal fly ash (solid sampling system).

Step	Parameters				Ar gas
	Temperature (°C)	Rate (°C s^−1^)	Hold time (s)	Total time (s)	
Preheating	70	1	60	114	Min
Drying	105	10	30	33.5	Min
Drying	120	10	20	21.5	Min
Ashing	600	100	20	24.8	Max
Ashing	1200	100	20	26.0	Middle
Ashing	500	100	50	57.0	Max
Autozero (AZ)	500	0	6	6.0	Middle
Atomization	2150	500	7	10.3	Middle
Cleaning	2600	1000	15	15.5	Max

**Table 3:** Optimization of the furnace program for the analysis of raw coal (liquid sampling system).

Integration time (s)	Pyrolysis temperature(s)	Rate (°C s^−1^)	Hold time (s)	Ar gas (L min^−1^)	Atomizer temperature (°C)	Rate (°C s^−1^)	Hold time (s)	Ar gas (L min^−1^)	Correlation coefficients
4.5	1200	100	60	Max	2600	1000	4	Mid	0.9705
6.0	1500	100	30	Max	2500	1000	6	Stop	0.9960

Mid: 1.0 L min^−1^, Max: 2.0 L min^−1^.

**Table 4:** Optimized furnace program for the analysis of raw coal (liquid sampling system).

Step	Parameters				Ar gas
	Temperature (°C)	Rate (°C s^−1^)	Hold time (s)	Total time (s)	
Drying	90	30	5	7.3	Max
Drying	105	10	20	21.5	Max
Drying	120	10	5	6.5	Max
Pyrolysis	1500	100	30	43.8	Max
Autozero (AZ)	1500	0	6	6.0	Stop
Atomization	2500	1000	6	7.0	Stop
Cleaning	2600	1000	10	10.1	Max

**Table 5:** Arsenic concentration (mg kg^−1^).

	As content			
Samples code	Raw coal (mg L^−1^)*	Fly ash (mg kg^−1^)**		
		FA EP 1	FA EP 2	FA EP 3
E	1.58	9.34	26.27	39.12
F***	1.37	10.55	12.13	27.03
G***	1.37	42.94	54.42	57.85
H****	4.2	9.22	23.51	28.29
I****	4.2	40.85	42.78	45.05
J	2.65	18.57	27.82	41.02
K	1.34	10.96	14.23	16.12

*Five raw coal samples.

**Twenty-one fly ash samples.

***F and G were the same raw coal samples.

****H and I were the same raw coal samples.

**Table 6:** Mass balance data for arsenic (%).

Sample	Mass balance (%) of As
E	113.2
F	112.3
G	113.3
H	110.7
I	101.9
J	101.8
K	119.0
